# Towards a Molecular Understanding of the Biosynthesis of Amaryllidaceae Alkaloids in Support of Their Expanding Medical Use

**DOI:** 10.3390/ijms140611713

**Published:** 2013-05-31

**Authors:** Adam M. Takos, Fred Rook

**Affiliations:** Plant Biochemistry Laboratory, Department of Plant and Environmental Sciences, University of Copenhagen, Thorvaldsensvej 40, 1871 Frederiksberg, Denmark; E-Mail: adta@life.ku.dk

**Keywords:** Amaryllidaceae, alkaloids, galanthamine, lycorine, narciclasine, *Narcissus*, *Galanthus*, *Lycoris*

## Abstract

The alkaloids characteristically produced by the subfamily Amaryllidoideae of the Amaryllidaceae, bulbous plant species that include well know genera such as *Narcissus* (daffodils) and *Galanthus* (snowdrops), are a source of new pharmaceutical compounds. Presently, only the Amaryllidaceae alkaloid galanthamine, an acetylcholinesterase inhibitor used to treat symptoms of Alzheimer’s disease, is produced commercially as a drug from cultivated plants. However, several Amaryllidaceae alkaloids have shown great promise as anti-cancer drugs, but their further clinical development is restricted by their limited commercial availability. Amaryllidaceae species have a long history of cultivation and breeding as ornamental bulbs, and phytochemical research has focussed on the diversity in alkaloid content and composition. In contrast to the available pharmacological and phytochemical data, ecological, physiological and molecular aspects of the Amaryllidaceae and their alkaloids are much less explored and the identity of the alkaloid biosynthetic genes is presently unknown. An improved molecular understanding of Amaryllidaceae alkaloid biosynthesis would greatly benefit the rational design of breeding programs to produce cultivars optimised for the production of pharmaceutical compounds and enable biotechnology based approaches.

## 1. Introduction

The chemically diverse alkaloids represent a large group of plant defence compounds which are characterised by containing a nitrogen atom in a heterocyclic ring [1]. Approximately 12,000 different alkaloids distributed over a number of distinct classes have been identified in plants, and several of these plant bioactive compounds have striking beneficial effects in the treatment of human medical conditions. The biosynthetic pathways for these different classes of alkaloids often start from amino acid derived precursors but have independent evolutionary origins. For example, the monoterpenoid indole alkaloids, which includes the anti-cancer drugs vinblastine and vincristine, are based on the condensation product of a monoterpenoid compound and an indole moiety produced from tryptophan. Opiates such as morphine and codeine are benzylisoquinoline alkaloids and produced following the condensation of two tyrosine-derived precursors. The biosynthesis of tropane alkaloids, such as nicotine and scopolamine, starts from the non-protein amino acid ornithine. However, purine alkaloids, such as caffeine, are not derived from amino acids but from purine nucleotides [1].

Given the medical importance of pharmaceutically active alkaloids, much research effort is focussed on establishing or improving their commercially viable production. Often compounds of pharmaceutical interest are present in low concentrations, or only present in specific tissues, or the availability of plant material is limited because a plant species may be difficult to cultivate, while collection from the wild is unsustainable. The research efforts to support commercial production include organic chemistry approaches to obtain partial or full chemical synthesis, production in cell or tissue culture, domestication and improved cultivation methods, development of new cultivars, and molecular methods to identify the biosynthetic genes and potentially establish production in heterologous systems [2–4].

Due to their complex stereochemistry, the organic synthesis of alkaloids of pharmaceutical interest is often highly challenging and consequently, not always economically viable. For instance, morphine was first chemically synthesized by Gates and Tschudi in 1952 and many additional partial and full synthesis methods have been developed since [5,6]. These efforts have greatly advanced the art of organic synthesis, but also lead to the realisation that a purely synthetic production process for morphine is unlikely to be competitive with production based on the cultivation of *Papaver somniferum*, the opium poppy [7]. In cases when the cultivation of the alkaloid producing plant is difficult or not practically possible, then cell cultures can be a sustainable production system although the costs associated with their maintenance are high. The best know example of this is probably the plant cell based commercial production of paclitaxel, better known as taxol, which was originally identified in the bark of the Pacific yew tree (*Taxus brevifolia*) [2,8].

**Figure 1:**
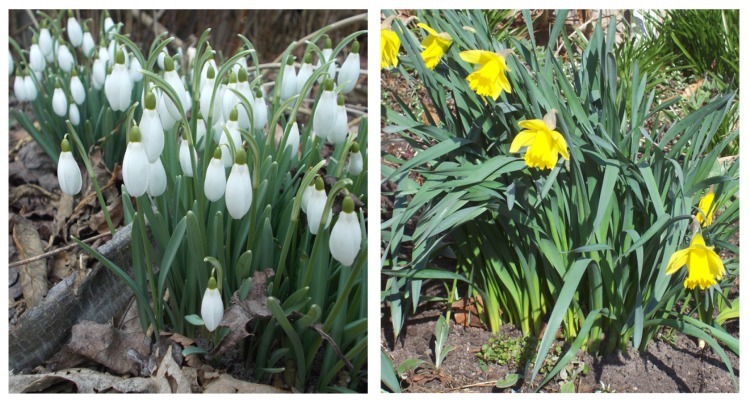
Amaryllidaceae species from genera such as *Galanthus* (snowdrops, **left**) and *Narcissus* (daffodils, **right**) are popular as ornamental plants in gardens.

Here we focus on the alkaloids characteristically produced by species of the Amaryllidaceae plant family as a class of alkaloids with promising medicinal potential. The Amaryllidaceae are a family of bulbous plants that includes well-known ornamental species such as those in the type genus *Amaryllis*, and genera like *Narcissus* (daffodils) and *Galanthus* (snowdrops) (Figure 1). At present the Amaryllidaceae alkaloids are primarily known for the clinical use of galanthamine in the symptomatic treatment of Alzheimer’s disease [9]. Although galanthamine can be produced by organic synthesis, plants are the main source for the pharmaceutical industry [10]. While extensively cultivated and bred for their ornamental flowers, species such as *Narcissus* spp., *Leucojum aestivum*, *Ungernia victoris* and *Lycoris radiata* are also grown for the commercial production of galanthamine [11]. The potential utility of these species is not just restricted to galanthamine, the Amaryllidaceae synthesize a diverse array of alkaloids with pharmacological activities, and compounds like lycorine, haemanthamine, tazettine and narciclasine are known for their anti-cancer properties [12–15]. Further clinical development and pharmaceutical application of Amaryllidaceae alkaloids will in part depend on establishing the sustainable production and commercial availability of alkaloids in addition to galanthamine. This will require research efforts such as the development of new cultivars with higher alkaloids content or improved alkaloid composition. Presently, the biosynthesis of Amaryllidaceae alkaloids is only partly characterised at a biochemical level, but no biosynthetic genes have been identified so far. An improved molecular understanding of Amaryllidaceae alkaloid biosynthesis will both rationalise breeding efforts and pave the way for biotechnological approaches to improve commercial production and availability. Here we review aspects of the Amaryllidaceae and their alkaloids ranging from chemical ecology to commercial production and discuss research challenges and opportunities.

## 2. The Amaryllidaceae Family and Their Medicinal Alkaloids

### 2.1. The Extent of the Amaryllidaceae Family

The Amaryllidaceae are a family of bulbous flowering plants in the monocot order Asparagales, and named after the type genus *Amaryllis*. There are evolving views regarding the extent of this family, and successive changes have been made to its status by the Angiosperm Phylogeny Group (APG). Most recently in APG III the Amaryllidaceae family was expanded, with the initial plant group with that name demoted to a subfamily named Amaryllidoideae [16]. In this new classification the wider Amaryllidaceae (Amaryllidaceae *sensu lato*) also include the subfamily Agapanthoideae, containing a single genus named *Agapanthus*, and the subfamily Allioideae. The type genus of the Allioideae, previously the family Alliaceae, is *Allium* which contains a large number of species of onions, garlics and leeks. However, the concept of Amaryllidaceae alkaloids is deeply embedded in the phytochemical and pharmaceutical literature, and the occurrence of this type of alkaloids is a characteristic of the original Amaryllidaceae family. Alkaloids are generally thought not to occur in the Agapanthoideae and Allioideae subfamilies, with the exception of a single report of unrelated canthinone type alkaloids, likely derived from tryptophan, in *Allium neapolitanum*, [17]. Therefore, continued use of the term Amaryllidaceae alkaloids to specifically mean the characteristic alkaloids found in the well-defined subfamily Amaryllidoideae, also given the uncertainty of future reclassifications, seems most practical. This more limited group of plants contains approximately 60 genera and over 850 known species.

Amaryllidaceae genera frequently mentioned in relation to traditional medicinal use and present drug discovery efforts include *Galanthus*, *Leucojum*, *Narcissus*, *Crinum*, *Lycoris*, *Clivia*, *Haemanthus*, *Pancratium* and *Hippeastrum* [18–25]. Often, species from these genera have been extensively cultivated for ornamental use due to their impressive floral displays. For example, *Crinum* species produce umbels of lily-like flowers and have a history of traditional medicinal use [21,26]. The genus *Lycoris* consists of approximately 20 species and has its natural distribution in East Asia. *Lycoris* species have been cultivated as ornamentals in China and Japan for many centuries, while *Lycoris radiata* (red spider lily) is used in traditional Chinese medicine and in the present commercial production of galanthamine [27,28]. Species of the genus *Galanthus* (snowdrops) were an early commercial source of galanthamine and are popular in gardens as a first sign of spring. Species from the genus *Leucojum* are known as snowflakes, *Leucojum aestivum* is an industrial source for galanthamine production in Eastern Europe, but collection from the wild has endangered natural populations [11,29]. The genus *Narcissus* is native to Europe and North-Africa, with its centre of biodiversity found on the Iberian Peninsula [30]. Due to the existence of natural hybrids, extensive cultivation and breeding, and escape and naturalisation, the number of recognised species of *Narcissus* varies from 26 to circa 80. Breeding of *Narcissus* cultivars has traditionally been concentrated in Britain and The Netherlands, and thousands of cultivars have been produced. Size, flower shape, and colour have been the main selection criteria in breeding new varieties. The Royal Horticultural Society classifies Narcissus cultivars in 13 divisions, and has registered more than 27,000 distinct varieties [31]. Because of it wide commercial availability and vigour, the variety *Narcissus pseudonarcissus* cv. Carlton is presently used in the commercial production of galanthamine.

### 2.2. Amaryllidaceae Alkaloids

**Figure 2:**
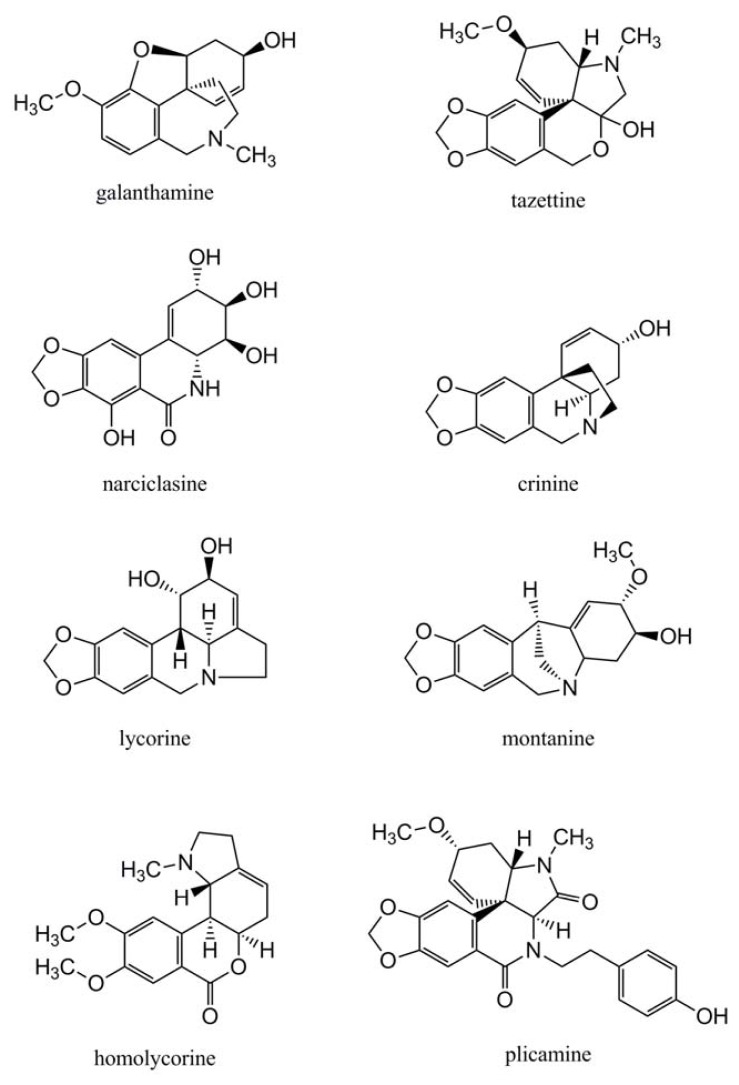
Chemical structures of Amaryllidaceae alkaloids mentioned in the text, illustrating some of the different subgroups.

The Amaryllidoideae are characterised by the presence of a biogenetically related group of alkaloids derived from norbelladine and over 300 different alkaloids have presently been identified from species of this subfamily [32]. Their common feature is a ring system composed of a C_6_–C_1_ unit derived from phenylalanine, and a N–C_2_–C_6_ unit derived from tyrosine. The alkaloids are classified according to their main skeleton structure and named after a representative alkaloid from the class. Well-known subgroups are, for example, the lycorine-type, the galanthamine-type, the tazettine-type and the narciclasine-type (Figure 2). Unfortunately, there is no strict consensus about the number of classes or their names. As the number of compounds discovered increases, new skeletal structures are being discovered, while the preference for classes and names may be influenced by the alkaloids present in the particular species under investigation.

Ghosal *et al.* recognised 12 distinct ring types when reviewing the alkaloids of the genus *Crinum* [33], a classification also followed by Evidente and Kornienko [12]. Unver introduced two new subgroups called the graciline-type and the plicamine-type, the latter containing two nitrogen atoms (Figure 2) [34]. Bastida and co-workers mostly focussed on *Narcissus* spp. and recognised nine principle subgroups [32]. Jin distinguished 18 subgroups of Amaryllidaceae alkaloids and this expansion is mainly due to the continued isolation of alkaloids with rare skeleton types from Amaryllidaceae species, but arguable also by the inclusion of alkaloids not identified within the Amaryllidaceae [35].

On three occasions, Amaryllidaceae-type alkaloids have been reported from species not belonging to the Amaryllidaceae. An early report of the isolation of lycorine and acetylcaranine from *Urginea altissima* (Hyacinthaceae) could not be confirmed and has been questioned [36,37]. More notably, Mulholland *et al*. [38] reported the isolation of crinamine from the tubers of *Dioscorea dregeana*, a species of yam belonging to the family Dioscoreaceae. This unexpected occurrence of crinamine is quite possibly the result of convergent evolution, a frequent phenomenon in plant specialised metabolism and known examples include alkaloid biosynthetic pathways [39–45]. *D. dregeana* also produces the alkaloid dioscorine, which has nicotinic acid as a precursor [46]. More recently, Amaryllidaceae-type alkaloids, such as lycorine and haemanthamine, were isolated from *Hosta plantaginea*, which also contained several new alkaloids, such as hostasine [47]. Of these, hostasinine A represented a new skeleton type [48], but its subsequent inclusion as an additional structure type of the Amaryllidaceae alkaloids [35] is however debatable, as strictly speaking, it has not been identified in an Amaryllidaceae species. Following a reclassification in APG III, the *Hosta* genus has been placed in the extended family of Asparagaceae [16], which, like the Amaryllidaceae *sensu lato*, belongs to the order Asparagales. The lack of additional reports for the wider occurrence of Amaryllidaceae-type alkaloids in the Asparagaceae and the evolutionary distance between *H. plantaginea* and the Amaryllidaceae also in this case suggest convergent evolution, but further detailed molecular analysis is required to conclusively resolve this issue.

### 2.3. Galanthamine in the Treatment of Alzheimer’s Disease and Other Neurological Conditions

The interest in the pharmaceutical applications of Amaryllidaceae alkaloids largely originates from the use of the alkaloid galanthamine, isolated from several species of Amaryllidaceae, in the symptomatic treatment of Alzheimer’s disease [9,49]. Experimental studies on galanthamine started in the Soviet Union and Bulgaria in the 1950s, but its origins may lay in traditional medicinal use of *Galanthus* species (snowdrops) to ease nerve pain and prevent permanent paralysis from poliomyelitis (reviewed in [9]). The discoveries that galanthamine was a reversible inhibitor of acetylcholinesterase, and that it could cross the blood-brain barrier, let to its clinical use for neurological conditions. In the 1960s and 1970s, the drug, produced from *Galanthus nivalis* and commercialised under the name Nivalin, was used in eastern European countries primarily to treat polio as it increased neurotransmission in the brain. Because of the political situation of the Cold War, and due to its limited availability from natural sources, galathamine only became more widely recognised for its potential in the 1980s. Clinical trials for use in Alzheimer’s disease were conducted in the 1990s, and the drug, now commercialised under the name Reminyl by Janssen Pharmaceuticals, part of Johnson & Johnson, was approved by the U.S. Food and Drug Administration (FDA) in 2001 for the treatment of mild to moderate cases of Alzheimer's disease. Reminyl was renamed Razadyne in 2005 to help avoid confusion with the diabetes drug Amaryl. Generic versions of the drug produced by over 15 drug manufacturers have now been approved by the FDA [50].

Application of galanthamine as a drug to treat conditions other than Alzheimer’s disease where cognitive functioning is impaired, or in relation to recovery from addiction, are active areas of medical research. For instance, galanthamine is not only recognised as an inhibitor of acetylcholinesterase, but also as an allosteric modulator of the neuronal nicotinic acetylcholine receptors (nAChRs) to which acetylcholine binds [51]. Galanthamine has been shown, both in *in vitro* and *in vivo* models, to have a neuroprotective effect on brain tissues subjected to, for instance, oxidative stress, or subjected to oxygen and glucose deprivation [52]. Galanthamine also acted as a neuroprotective agent on *in vivo* models when administered within three hours following an episode of reduced blood flow to the brain (cerebral ischemia), aiding in memory recovery, and this effect was mediated by nAChRs [53]. This protective effect of galanthamine may have therapeutic potential for preventing neuronal death following a stroke, and is presently relevant in a clinical context for patients with mixed or vascular dementia (resulting from vascular disease). Similarly, galanthamine is an effective antidote against poisoning with organophosphorus compounds (OPs) such as pesticides and nerve agents (e.g., soman and VX), counteracting both the acute toxicity as well as OP-induced neurodegeneration [54,55]. Both the projected increase in the number of patients with Alzheimer’s disease, due to an aging population [56,57], as well as usage of galanthamine to treat other medical conditions, is likely to result in an increased demand for the compound.

### 2.4. Amaryllidaceae Alkaloids and Their Pharmacological Activities

A substantial number of additional pharmacological activities have been described for Amaryllidaceae alkaloids, which we will only review briefly. Most notably, several Amaryllidaceae alkaloids are being evaluated as anti-cancer drugs [12]. Lycorine is a powerful inhibitor of ascorbic acid biosynthesis, and also a powerful inhibitor of cell growth and cell division, including antitumor activity in animal and human cell lines [58,59]. Other alkaloids of this type, such as caranine, galanthine, pseudolycorine and 2-*O*-acetylpseudolycorine, are also active against a variety of tumour cells. Narciclasine is showing highly promising anti-cancer effects against human glioblastoma multiforme (GBM) tumours in preclinical animal models [13]. This most malignant type of primary brain tumour is characterised by aggressive invasive behaviour into normal brain tissues, and resistance to conventional therapies that trigger apoptosis. Narciclasine impaired the growth of GBM tumours and significantly extended the survival time of immunodeficient mice with xenografts of GBM tumours into their brain [13,14]. These experiments also demonstrated the ability of narciclasine to cross the blood-brain barrier, a characteristic often lacking in many drugs. More, recently, cytostatic activity of a crinine-type Amaryllidaceae alkaloid, bulbispermine, against a panel of apoptosis resistant brain cancer cells lines was reported [60]. Lycorine and pseudolycorine have antiviral activities against a number of RNA and DNA-viruses. Several Amaryllidaceae alkaloids show activity against protozoan parasites such as *Plasmodium falciparum*, the cause of malaria, and *Trichomonas vaginalis*, the cause of the sexually transmitted disease trichomonosis [61,62].

## 3. Ecological and Physiological Aspects of Amaryllidaceae Alkaloids

### 3.1. Chemical Ecology of Amaryllidaceae Alkaloids

In contrast to the extensive literature on the phytochemistry and pharmaceutical applications of Amaryllidaceae alkaloids, their natural physiology and ecological role is much less investigated. For example, their effects on natural occurring insect herbivores or microbial pathogens are largely unreported. In the case of galanthamine it can be argued that its target enzyme acetylcholinesterase is also the principle target for most commercially available insecticides [63]. Similar to insecticides, galanthamine is likely to provide the plant with some level of protection against pests with a nervous system. Given the economic importance of insecticides, insect resistance against organophosphate or carbamate pesticides has been investigated extensively, and resistance frequently resulted from differences in sensitivity or mutations in acetylcholinesterases [64,65].

For example, a recent study on the genetic variation in the Colorado potato beetle (*Leptinotarsa decemlineata*), an invasive species and a major threat to potato crops worldwide, showed that the frequency of an insecticide resistance-associated mutation in an acetylcholinesterase gene was very high in the native Mexican population and therefore its likely ancestral state [66]. The authors suggested that this reflected the beetles’ adaptation to the high levels of steroidal alkaloids found in its wild Solanaceous food plants, pre-adapting the insect to organophosphate insecticides. Similarly for plant-insect interactions involving Amaryllidaceae alkaloids, it was shown that the main alkaloid from *Hippeastrum puniceum*, 3-*O*-acetyl-narcissidine, is an antifeedant against the generalist herbivore *Spodoptera littoralis* (African Cotton Leafworm), but not against the above mentioned more specialised *L. decemlineata* [67]. In contrast to *S. littoralis*, a few other moths belonging to the Noctuidae family have specialised on Amaryllidaceae: *Polytela gloriosa* (Indian Lily Moth) is known to sequester alkaloids, such as lycorine, from its food plants [68], while the larvae of *Brithys crini* (or Lily Borer) are also known to be toxic. Some insects infesting *Narcissus* species in a natural context have also been reported. The scathophagid fly *Norellia melaleuca* lays a single egg inside the flower bud and the fly larva tunnels its way through the pistil and the ovary to eventually pupate within the hollow flower stem [69].

There are several suggestions that some Amaryllidaceae alkaloids can act as allelopathic chemicals, repressing the growth of other plants. For example, *Lycoris radiata* is used as a traditional ground covering plant on the levees of rice paddy fields, preventing soil erosion and suppressing weeds [70]. Soil mixed with leaf tissue of *L. radiata* was able to repress seedling emergence and growth of several plant species, and lycorine was identified as one of the potential allelochemicals. An additional suggestion for the role of lycorine in plant-plant interactions is provided by an unusual report on the penetration of the bulbs of *Pancratium biflorum* by roots of the ecological invasive weed *Imperata cylindrica* (cogongrass) [71]. This resulted in a hypersensitivity response with necrosis of the bulb tissue surrounding the penetrating root and the accumulation of polyphenolic compounds and changes in alkaloid composition. Healthy scales of *P. biflorum* contained lycorine-1-*O-*β-d-glucoside and lycorine-1-*O-*(6′-*O*-palmitoyl-β-glucoside) as major compounds, but no free lycorine. In contrast, the necrotic tissue contained abundant quantities of free lycorine, likely as part of a defence response. Antimitotic effects of exogenously applied narciclasine on plant growth have been reported as early as 1967 [72], and although there is molecular specificity to its effects, the more recent discussion of the broad cytotoxic effects of narciclasine in the context of a role as a plant growth regulator for which a signal transduction pathway can be dissected, seems an incorrect use of terminology [73,74]. In general, although plant growth inhibition by phytotoxic compounds, such as several Amaryllidaceae alkaloids can be shown, proving an allelopathic role in a natural setting is more challenging as it is often unknown if these compounds are exuded by the plant into the soil [70].

A particular relevant research question is also how Amaryllidaceae species avoid self-toxicity. Sequestration of toxic defence compounds into specialised cell types or anatomical structures, such as idioblast, laticifers and resin ducts, is one type of adaptation frequently observed in plant chemical defense, the latex and alkaloid containing laticifers of opium poppy being a well-known example [1]. The release of an irritant mucous sap from damaged bulbs or cut stems and leaves of species, such as *Narcissus*, is well known in the flower bulb industry and the cause of a dermatological condition called “lily rash” [75,76]. The mucus contains calcium oxalate crystals, which provide protection against herbivory [77], but is also rich in alkaloids. Anatomical studies of the Amaryllidaceae are surprisingly rare, but mucus filled cavities separated by vascular bundles have been described for the leaves of *Galanthus* and *Leucojum* species [78], and “slime vessels” have been mentioned for *Narcissus* flower stems and are disrupted during flower picking [76]. It was suggested that these mucus containing structures form by cell disintegration during development. With possible alkaloid sequestration in specialised anatomical structures come further questions related to the cellular localisation and timing of alkaloid biosynthetic gene expression, and the transport of alkaloids or their intermediates [1]. It is therefore of interest that alkaloid glycosides, such as lycorine-1-*O*-β-d-glucoside in *P. biflorum*, have been reported to commonly occur in several Amaryllidaceae species [79]. These seem to be produced during specific times in development, such as during flowering and periods of great activity. One possible explanation here is that such glycosylated alkaloids could be transport forms of the free alkaloid, increasing solubility, but perhaps also avoiding self-toxicity during transport.

### 3.2. Physiology of Amaryllidaceae Alkaloids

Central to the overall physiology of the Amaryllidaceae is that they are geophytes: perennial plants containing an underground storage organ, and a life cycle that includes a dormancy period [80,81]. Starch accumulated during the growth season is the main storage carbohydrate in the bulb and serves to support the plant’s metabolism during its dormancy period, often an unfavourable dry or cold period, and enables rapid flowering and sexual reproduction at the start of the new growth season [82]. The high alkaloid content of the bulbs primarily serves to protect the plant’s carbohydrate resources from herbivores and microbial organisms [83]. The breaking of dormancy of seeds and bulbs often requires a cold period, and in the commercial flower bulb industry such cold treatments are often artificially controlled to precisely time the date of flowering [84,85]. Physiological research on ornamental bulbs has focussed on optimising their cultivation and propagation to deliver high quality bulbs and flowers [86,87].

Research specifically into the physiology of Amaryllidaceae alkaloids mostly aims to improve alkaloid production in a galanthamine producing crop such as *Narcissus* spp. or *Lycoris* spp. Tissue specific distribution, optimal time of harvest, treatments fertilizer or pesticides, and the effects of growth media and plant hormones on cultured tissues, are some of the main focal points [10,88–91]. An NMR-based study on the effect of fertilizers on galanthamine content in *N. pseudonarcissus* cv. Carlton concluded that application of the standard fertilizer treatment used in the ornamental bulb industry caused a significant increase in galanthamine content compared to a non-treated control, and was sufficient for optimal galanthamine production [10]. The present cultivation of ornamental flower bulbs is characterised by a high input of agrochemicals, for instance to prevent fungal diseases. Lubbe *et al*. (2012) investigated the effect of various fungicide treatments typically used in *Narcissus* cultivation on the content of galanthamine and other metabolites in *N. pseudonarcissus* cv. Carlton after harvesting [88]. Bulbs that had received a fungicide treatment before planting mostly showed an average galanthamine content similar to an untreated control. In contrast, several foliar fungicide treatments applied in the field resulted in reduced galanthamine content and an increase in soluble sugars such as sucrose and glucose. Alkaloid content in Carlton varied during the growth season and was highest before flowering, but harvest at the end of the season was recommended because of a higher bulb biomass available for extraction and the possibility to select bulbs for replanting for the following season [91].

In *L. aurea*, galanthamine content was lowest in leaves and highest in bulbs on a fresh weight basis. Of the total amount of galanthamine present, 99.5% was present in bulbs and roots based on both fresh and dry weight basis [89]. The highest galanthamine content in roots and bulbs was observed in May and June, respectively, with leaves withering in May in this autumn flowering species. The galanthamine levels in bulbs steadily declined by over 50% in subsequent months, reaching their lowest levels in October following leaf emergence in September. *L. aurea* responded adversely to high nitrogen application, leading to dramatic reductions of total leaf area, dry weight, and galanthamine content of plant tissues [89]. The bulbs in this study contained a substantial amount of nitrogen before root emergence, and the best results were obtained in the absence of any additional nitrogen.

## 4. A Biochemical and Molecular Understanding of Amaryllidaceae Alkaloid Biosynthesis

### 4.1. Present Knowledge on Amaryllidaceae Alkaloid Biosynthesis

**Figure 3:**
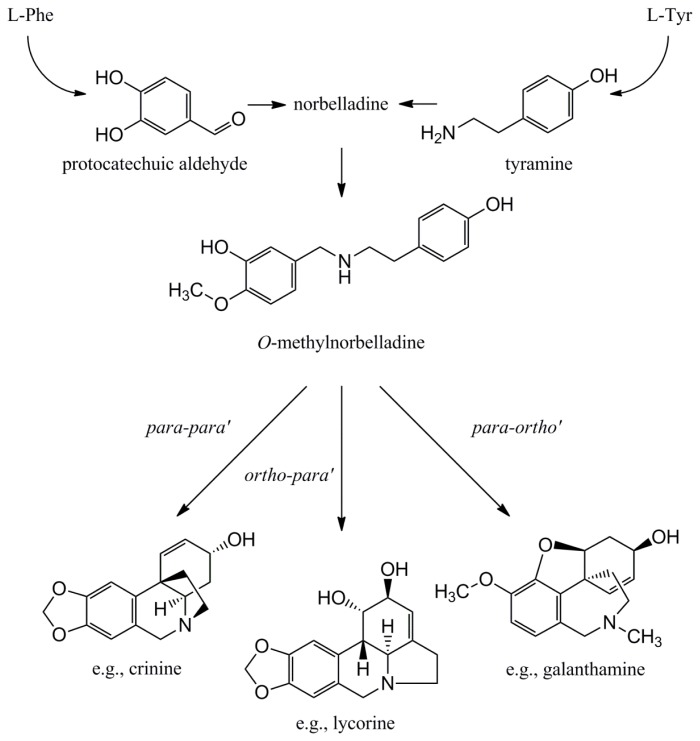
Schematic overview of Amaryllidaceae biosynthesis. Starting from the aromatic amino acids phenylalanine and tyrosine the common precursor *O*-methylnorbelladine is formed. Alternative ways of oxidative phenol coupling lead to the various Amaryllidaceae alkaloid skeleton types.

The biosynthesis of Amaryllidaceae alkaloids has been investigated biochemically using labelled precursors and intermediates, and biochemical scenarios for the synthesis of the various types of alkaloids have been proposed (reviewed in [20,32]). Most of this research was conducted in the 1950s and 60s, and a variety of Amaryllidaceae species and cultivars were used by the different research groups. Although their conclusions are now often generalised, the existence of subtle differences in the biosynthesis of a specific alkaloid between species cannot be ruled out [92–94]. Amaryllidaceae alkaloids are derived from the aromatic amino acids phenylalanine and tyrosine, which are used to produce the common precursor *O*-methylnorbelladine. Alternative ways of oxidative phenol coupling produce three main skeleton types that form the basis of further alkaloid diversity in the Amaryllidaceae (Figure 3). A complex network of enzymatic steps with one alkaloid acting as the precursor to another, produces a spectrum of compounds that differs between species and cultivars, and even between the different tissues of the same plant [20,32]. Each Amaryllidaceae species produces a mixture of alkaloids, often with a few dominant compounds and a larger number of compounds at lower concentrations, likely to result from differences in the substrate specificity and expression level of the various biosynthetic enzymes present. Although the classes of biosynthetic enzymes involved can largely be predicted, surprisingly no Amaryllidaceae alkaloid biosynthetic genes have been identified or characterised to date, and a molecular genetic understanding of alkaloid production is lacking. Understanding which combination of genes results in which alkaloids would be highly beneficial to the rational design of breeding programs, and to enable metabolic engineering.

#### 4.1.1. Initial Biosynthetic Steps

The biosynthesis of Amaryllidace alkaloids, as is usually the case in plant specialized metabolism, starts with the recruitment of substrates and enzymes from general metabolism. The enzyme phenylalanine ammonia lyase (PAL) converts phenylalanine into cinnamic acid and ammonia, while tyrosine is decarboxylated by tyrosine decarboxylase to yield tyramine. Cinnamic acid is degraded further to give protocatechuic aldehyde and its condensation with tyramine results in a Schiff base intermediate [95], which following reduction results in the formation of norbelladine and its derivatives (Figure 3). The enzymes catalysing these last steps are unknown and a comparison to other alkaloid biosynthetic pathways suggests that their molecular identity may be difficult to predict.

**Figure 4:**
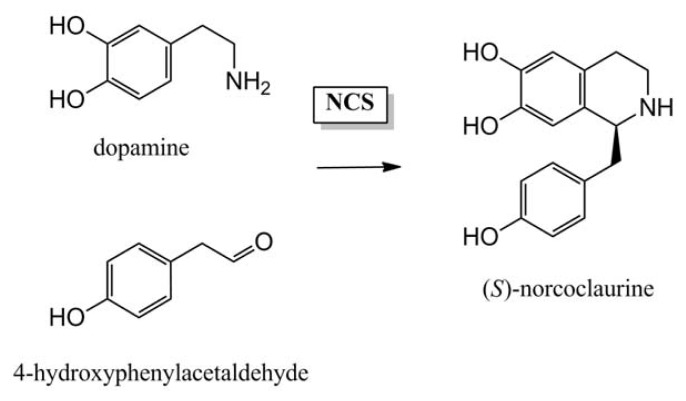
Formation of (*S*)-norcoclaurine in the biosynthesis of benzylisoquinoline alkaloids by a Pictet-Spengler reaction. The condensation reaction between the two tyrosine derived precursors dopamine and 4-hydroxyphenylacetaldehyde is catalysed by norcoclaurine synthase (NCS).

In the synthesis of benzylisoquinoline alkaloids in the Ranunculaceae and Papaveraceae plant families, specifically in species such as *Thalictrum flavum* and *Papaver somniferum* respectively, the condensation of dopamine and 4-hydroxyphenylacetaldehyde to norcoclaurine is mediated by the enzyme norcoclaurine synthase. This enzyme catalyses a Pictet-Spengler condensation by a two-step reaction mechanism that involves a condensation step followed by an intramolecular cyclisation [96] (Figure 4). The fact that the electron-donating oxygen of the hydroxyl group at C-2 of dopamine, not present in tyramine, is essential for this reaction to proceed provides a mechanistic explanation for the absence of cyclisation in the condensation step of Amaryllidaceae alkaloid biosynthesis. Norcoclaurine synthases show their highest sequence homology with the Bet v1/PR10 protein family [97,98]. For the monoterpenoid indole alkaloids synthesized by for instance *Catharanthus roseus* and *Rauvolfia serpentina* (family Apocynaceae), strictosidine is the precursor and formed by a Pictet-Spengler condensation of tryptamine and secologanin [99]. Strictosidine synthase is a member of the six-bladed β-propeller fold protein family unique to plants [100,101]. These examples show that the initial condensation reactions in alkaloid biosynthesis can be catalysed by members of very different protein families, and suggests that the type of enzyme recruited for the non-cyclising condensation reaction in the Amaryllidaceae may well belong to an additional class of enzymes.

From an evolutionary perspective it is of interest that the alkaloid precursors tyramine and protocatechuic aldehyde are bioactive compounds in their own right. Protocatechuic aldehyde is a phenolic compound with antioxidant and antimicrobial activities found in many plants [102,103]. Tyramine is known in neurochemistry as a trace amine, having a function as a neurotransmitter in invertebrates and interacting with G-protein-linked trace amine associated receptors (TAARs) in mammals [104,105]. Tyramine is also the direct precursor for tyramine-type protoalkaloids, such as hordenine (*N*,*N*-dimethyltyramine), and it is noteworthy that such protoalkaloids are the dominant compounds present in a large number of natural populations of *Galanthus elwesii* [106]. Alkaloids of the belladine type, compounds lacking the nitrogen containing heterocycle, have been found in the genus *Crinum* and are dominant compounds in two *Nerine* species [33,107,108]. These observations suggest that the more complex “true alkaloids” of the Amaryllidaceae could have evolved via adaptive intermediate steps.

#### 4.1.2. Phenol Coupling

A key biosynthetic step in the biosynthesis of Amaryllidaceae alkaloids is the cyclisation of *O*-methylnorbelladine by three alternative ways of C–C phenol coupling referred to as *ortho-para*, *para-para* and *para-ortho*, leading to Amaryllidaceae alkaloids with different core skeletons (Figure 3). For example, alkaloids of the galanthamine type are obtained from the *O*-methylnorbelladine precursor by a *para-ortho* phenol coupling step [94]. The enzymatic specificity of the phenol coupling step is difficult to reproduce by chemical synthesis, although progress has been made [109], and one of the reasons that the synthetic production of many of these compounds is highly challenging. Known examples of enzymes catalysing intramolecular phenol coupling reactions are cytochrome P450 enzymes.

In *C. japonica*, the enzyme that catalyses the intramolecular C–C coupling of two phenolic rings in the biosynthesis of the alkaloid (*S*)-corytuberine from (*S*)-reticuline was identified as CYP80G2 [110]. A similar intramolecular phenol coupling step in the morphine biosynthetic pathway in opium poppy is catalysed by a cytochrome P450 enzyme called salutaridine synthase, which was identified as a member of the CYP719 family named CYP719B1 [111]. CYP719B1 was highly selective for its natural substrate (*R*)-reticuline. Two human cytochrome P450 enzymes, P450 2D6 and P450 3A4, were able to take (*R*)-reticuline and (*S*)-reticuline as substrate and catalyze intramolecular phenol coupling reactions that yielded a number of different products, including salutaridine the *para-ortho* coupled precursor of morphine [112]. In the pathogenic bacterium *Mycobacterium tuberculosis*, the causative agent of tuberculosis, a cytochrome P450 named CYP121 catalysed the formation of an intramolecular C–C bond in a cyclodipeptide substrate [113].

The above examples demonstrate that specific cytochrome P450 enzymes are able to conduct an intramolecular C–C phenol coupling reaction, but that in plant alkaloid biosynthesis members of at least two CYP-families have acquired this ability. Recent progress in plant transcriptomic and genomic sequencing programs has provided insights into the evolution of plant cytochrome P450 families. The CYP71-clan contains many plant specific families of cytochrome P450 enzymes, many of them involved in plant specialized metabolism. Also, the CYP80 and CYP719 families belong to this CYP71-clan, but crucially for alkaloid biosynthesis in the Amaryllidaceae, neither family occurs in monocot plant species [114]. Although the phenol coupling steps in the Amaryllidaceae are likely to involve cytochrome P450 enzymes, these enzymes have independently evolved this ability and no candidate gene family can presently be suggested. While alkaloids from all three alternative ways of phenol coupling of the *O-*methylnorbelladine precursor can co-occur in a single plant, some species or cultivars will only contain one type [115], suggesting that multiple genes and/or different alleles for the phenol coupling enzyme exist.

#### 4.1.3. Further Skeleton Structures, Decorations and Modifications

Following the phenol coupling step, the three cyclisation products give rise to the different groups of Amaryllidaceae alkaloids and their further chemical diversity by various combinations of successive hydroxylations, oxidations and reductions, and methylation and demethylation reactions. Labelling studies have suggested intermediates and reaction mechanisms for a number of specific biosynthetic pathways, as well as their interactions and interdependencies. Here, we will highlight some general examples of interest, but for a detailed overview the reader is referred to [20,32].

**Figure 5:**
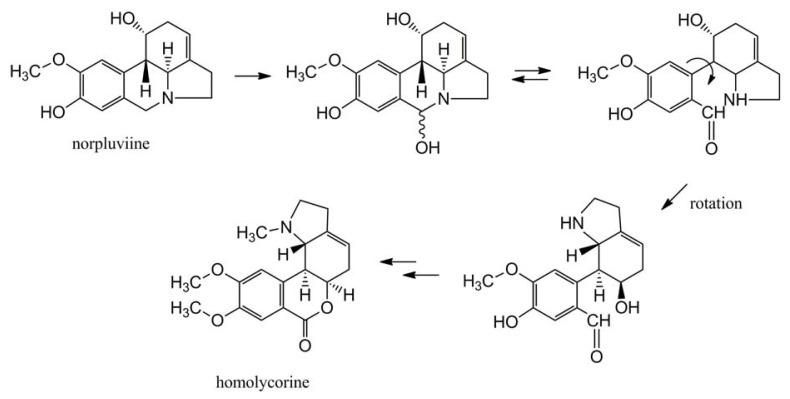
Formation of homolycorine from norpluviine following ring opening and intramolecular rotation.

The *ortho*-*para* phenolic coupling gives rise to both the lycorine and homolycorine groups of alkaloids via norpluviine. A reoxidation of the carbon atom in the central nitrogen containing ring, originally the aldehyde carbonyl carbon in protocatechuic aldehyde, leads to ring opening (Figure 5). Following an intramolecular rotation and hemiacetal formation, homolycorine type alkaloids are formed. In *Narcissus* cv. King Alfred, norpluviine is primarily converted to homolycorine type alkaloids [32], also providing a possible explaining why its descendent cv. Carlton produces homolycorine but not lycorine. This illustrates the competition between the formation of the different alkaloid groups.

A similar oxidation as described above starts from haemanthamine, itself a product of a *para-para* phenolic coupling. This oxidation results in an epimeric mixture of haemanthidine and epihaemanthidine, and following an intramolecular rotation, irreversibly to pretazettine [116]. As haemanthamine is a major compound found in *Narcissus* cv. Carlton, we can only speculate on the similarity between the enzymes responsible for the initial oxidations leading to either homolycorine or pretazettine. Of interest is also the biosynthesis of narciclasine as it structurally resembles both haemanthamine and lycorine which result from different phenolic couplings. Labelling studies have suggested that narciclasine is formed via a *para-para* phenol coupling, with 11-hydroxyvittatine as intermediate [32]. This compound was also proposed as intermediate in the biosynthesis of haemanthamine and montanine, again demonstrating the competition in the formation of the different alkaloid groups.

Of particular interest, given the pharmaceutical applications, has been the formation of galanthamine type alkaloids following a *para-ortho* phenol coupling reaction. Eichhorn *et al*. [94] used *Leucojum aestivum* as their experimental system and proposed that following the oxidative phenol coupling of *O*-methylnorbelladine, a spontaneous closure of an ether bridge would result in *N-*demethylnarwedine, which is subsequently reduced to norgalanthamine, and finally *N*-methylated to galanthamine. They also argued that narwedine was not a direct precursor of galanthamine as was previously proposed [93], but suggested that a reversible oxido-reductase could interconvert both compounds. Whether this proposed oxido-reductase is related to the one converting *N-*demethylnarwedine to norgalanthamine is an open question.

### 4.2. Gene Discovery Using Transcriptomics

Although the biochemical approaches with labelled intermediates described above have given detailed insights in many of the biosynthetic steps of Amaryllidaceae alkaloid production, molecular knowledge at the gene level is presently lacking. Such an understanding of the biosynthetic pathway at the molecular level would enable rational approaches to the optimisation of commercial alkaloid production. For example, by aiding the breeding of new cultivars with improved alkaloid levels or composition, but also pave the way for metabolic engineering, and for Amaryllidaceae alkaloid production in microbial hosts or alternative plant species. Transcriptome analysis, or even proteomic analyses, combined with metabolic profiling allows gene identification by correlating alkaloid production with the expression of specific genes (reviewed in [117]). Cell-type or tissue specific production of plant bioactive compounds, or induction of their biosynthesis following elicitor treatment, are some of the experimental conditions that allowed the identification of specific biosynthetic genes. An example of this approach is the methyl jasmonate induction of a *Taxus cuspidata* cell culture, which resulted in the identification of two cytochrome P450 cDNA clones that encoded hydroxylases of the taxol biosynthetic pathway [118]. More recent progress in DNA sequencing technology has resulted in several new initiatives to sequence the transcriptome of a substantial number of medicinal plant species, including members of the Amaryllidaceae [4,119,120].

Correlating the gene expression and alkaloid profiles of species or cultivars that differ in their alkaloid composition has also been used successfully to identify biosynthetic genes for specific alkaloids, for instance in *Papaver* spp. [119,121,122]. Recently 105 ornamental cultivars of *Narcissus* were analysed for their galanthamine content in both leaves and bulbs [115]. Galanthamine was absent in the bulbs of nine varieties, two only contained alkaloids in bulbs but not in leaves, and in one semi-dwarf cultivar galanthamine was the only alkaloid present. Also here, a comparative transcriptomic analysis may be used to identify candidate genes for specific biochemical steps in alkaloid biosynthesis.

### 4.3. The Potential Use of Biosynthetic Gene Clusters in Gene Discovery

Great potential for new ways of gene discovery is suggested by the observation that the non-homologous biosynthetic genes for several classes of plant chemical defence compounds are organised in genomic gene clusters [123,124]. In fungi, gene clusters for antibiotics or other pharmaceutical compounds of interest are common place, and strategies to elucidate their biosynthetic pathways often start from the characterisation of the genes found in specific gene clusters [125]. Such elucidation strategies based on gene clusters also hold great promise for the discovery of biosynthetic pathways for plant bioactive natural products. This was eloquently demonstrated for the first identification of a plant biosynthetic gene cluster for alkaloids. Winzer *et al.* [122] reported a gene cluster of 10 genes for the synthesis of the antitumor alkaloid noscapine in opium poppy (*Papaver somniferum*). Transcriptome analysis of the high noscapine variety HN1 identified a number of candidate biosynthetic genes, and genetic analysis of a F2 mapping population showed that these genes were tightly linked and associated with the presence of noscapine. The sequencing of a contig of bacterial artificial chromosomes revealed the presence of a gene cluster.

We recently reported the independent evolution of biosynthetic gene clusters for cyanogenic glucosides in three plant lineages [40], and have proposed a general evolutionary mechanism, based on antagonistic ecological selection pressures, that explains the formation of these genomic gene clusters [124]. Briefly, selection for reduced recombination between alternative beneficially interacting allele combinations, following randomly occurring genomic rearrangements, favours the ever closer physical linkage of the interacting loci. This preserves polymorphic traits that are under balancing selection in a natural population. A similar genetic mechanism is thought to contribute to the evolution of sex chromosomes under sexually antagonistic selection. In the case of plant chemical defence pathways, the alternative beneficial allele combinations may well be the presence or absence of a functional chemical defence pathway, and such natural occurring chemical defence polymorphisms are well described for cyanogenesis in populations of white clover (for a more detailed explanation see [124]).

Whether gene clusters also exist for Amaryllidaceae alkaloids is an important research question to address. The evolutionary model for gene cluster formation described above is based on the existence and maintenance of natural chemical defence polymorphisms, and there is evidence for the existence of such intraspecific and intrapopulation variation in alkaloid composition in Bulgarian populations of *Galanthus elwesiii*, *Galanthus nivalis* and *Leucojum aestivum* [29,106]. This suggests that the ecological conditions that are driving gene cluster formation, according to the model, could well be present. It is self-evident that the presence of gene clusters, and the resulting co-inheritance of specific combinations of biosynthetic genes, will have profound implications for breeding strategies that aim to improve or change alkaloid content and composition. In the case of the Amaryllidaceae, a technical challenge to isolate such clusters to aid gene identification will be their relatively large and uncharacterised genomes. For example, *Narcissus* species and cultivars are characterised by a great variety in their DNA content, also due to differences in ploidy levels. For diploids, the nuclear DNA content (2C) was shown to vary from 14 to 38 pg [126], which corresponds to an estimated haploid genome size of 6.8 to 18.6 Gb, or about 50–140 times the genome of the genetic model plant *Arabidopsis thaliana*.

## 5. Perspectives on Amaryllidaceae Alkaloid Production

Many bioactive natural compounds that have become of interest for pharmaceutical use are only available from natural sources in limited amount and finding reliable and economically viable production methods may involve different strategies [2,4]. Each strategy has its own challenges and which is the most pragmatic and economically viable one will largely depend on the compound in question and may change over time due to technological innovations, increase in knowledge, and economic and political developments [127–130]. Production from the original plant species may be complicated if it only produces a small amount of the desired compound or if the species is difficult to cultivate, taxol originally identified in the bark of the Pacific yew tree being the best known example of this [2]. Like for taxol, production based on tissue culture grown plant material or cell cultures can then be a solution, although such tissue culture production is often influenced by the differentiation state of the tissue and is relatively expensive due to their high cost of maintenance [2,131]. The economic viability of total chemical synthesis decreases the more chemically complex the molecule to be produced is, as every additional synthesis and purification step demands the commitment of more resources, reduces the overall yield, and increases the amount of chemical waste produced. Semi-synthesis starting from a complex biologically produced precursor can be more cost-effective and environmentally friendly, and such a combinatorial production process has been developed for the anti-malaria drug artemisinin [132–134]. Increasingly, the use of petrochemicals is becoming ecologically and politically undesirable and research and development policies, for example in Europe and the United States, have shifted to promoting sustainable and environmental friendly production methods as part of an envisaged bioeconomy [129,130,135].

Production of a specific bioactive compound in a heterologous host system, such as another plant species or a microorganism, will require that at least the most essential biosynthetic genes of the pathway have been identified. In the more recently advocated context of a synthetic biology approach, genes from different organisms can be combined in a heterologous host [136], for example the human phenol coupling enzyme P450 2D6, mentioned earlier in relation to endogenous morphine production, was used to convert (*R*)-reticuline to salutaridine in a yeast system [137]. To date, mostly partial pathways have been expressed in microorganisms such as *Escherichia coli* and the yeast *Saccharomyces cerevisiae*, while in plants engineering studies were meant to demonstrate proof-of-concept [138,139]. These studies have demonstrated that metabolic engineering of the host organism may be required to alleviate metabolic bottle necks in the compound’s production, to redirect the host’s metabolism away from competing endogenous biosynthetic pathways, or to prevent undesirable product conversions or the accumulation of toxic intermediates [4,128,140,141]. For example, production of artemisinic acid, a precursor for the antimalarial drug artemisinin, in the yeast *Saccharomyces cerevisiae* was optimised by engineering its mevalonate pathway [132]. In microorganisms and hetereologous plant production systems, gene silencing and plasmid or transgene instability, as well unexpected metabolic feedback mechanisms, were also among the problems encountered [141–143].

If plant cultivation on an industrial scale is possible, than the original plant may well be the physiologically and biochemically best-adapted production system. Apart from cultivar optimisation by traditional breeding, its metabolic engineering by transgenic approaches also has substantial potential, although politically more sensitive, particularly in Europe. For example, overexpression in *P. somniferum* of its cytochrome P450 enzyme CYP80B3, a (*S*)-*N*-methylcoclaurine 3′-hydroxylase acting in the morphine biosynthetic pathway upstream of the branch point intermediate (*S*)-reticuline, resulted in an up to 450% increase in the amount of total alkaloid found in latex [144]. Interestingly, *CYP80B3* overexpression resulted in a coordinated upregulation of other genes in the pathway, such as berberine bridge enzyme and codeinone reductase, again suggesting the importance of regulatory interactions [143,144]. Present commercial production of pharmaceutical alkaloids is discussed below and uses two of the best known examples, the alkaloids production in *Catharanthus roseus* and *Papaver somniferum*, as a guide for the most pragmatic strategies of Amaryllidaceae alkaloid production for the immediate and medium term future.

### 5.1. Examples of Present Commercial Alkaloid Production

The terpenoid-indole alkaloids vinblastine and vincristine are very powerful anticancer drugs that are produced in small quantities by the pantropical plant species *Catharanthus roseus*, which produces about 130 different alkaloid compounds [142]. Both intact plants and cell cultures of *C. roseus* grown in bioreactors are used to produce a number of compounds, but the cell cultures can only produce some types of alkaloids. Major areas of plant cultivation are found in the southern part of the USA, Mexico, South America, the Middle East, China and India. Breeding programs have been set up to modify alkaloid content in *C. roseus*. Vinblastine and vincristine are only present as minor compounds, while their precursors occur as major products in the plant. Both alkaloids can be produced from these precursors using semi-synthetic procedures, increasing overall production. The pharmaceutical company Eli Lilly produces vincristine by such a semi-synthetic process.

Morphine is produced by the opium poppy (*Papaver somniferum*) and used to relieve severe pain, although with the development of tolerance and dependencies. Opium poppy produces more than 50 different alkaloids, and special cultivars have been developed for the production of, for instance, thebaine [122], which is used for the semi-synthesis of such compounds, like oxycodone, and these cultivars produce very little morphine. The vast majority of legally produced morphine is still derived from the opium poppy itself, and for instance, the pharmaceutical company GlaxoSmithKline supplies 25% of the world’s medicinal opiate needs from poppies grown by farmers in Tasmania and has an active research program to produce new opium poppy varieties [144,145].

### 5.2. Amaryllidaceae Species and Present Commercial Galanthamine Production

Galanthamine is the only Amaryllidaceae alkaloid presently produced on a large commercial scale for the pharmaceutical industry. Other Amaryllidaceae alkaloids are only available in small quantities for research purposes, suggesting there is much untapped potential. Galanthamine was originally identified and extracted from natural populations of snowdrops (*Galanthus* spp.) from the Bulgarian mountains, but this was quickly replaced by snowflakes (*Leucojum* spp.), which have a higher biomass. It was this galanthamine from natural sources that was originally used for the development work of galanthamine based drugs for the treatment of Alzheimer’s. *L. aestivum*, collected from the wild, or commercially cultivated on small plantations, is still used for commercial galanthamine production in Eastern Europe [29]. The widespread licensing of galanthamine resulted in searches for new and more sustainable sources of the compound. This led to the genus *Narcissus*, as it was indigenous to Europe and plant material could be obtained in large quantities at a low price due to its cultivation as an ornamental plant [146]. Moreover, extensive knowledge on the propagation, physiology, breeding and cultivation of *Narcissus* existed in the commercial flower bulb industry. The popular *Narcissus* cultivar “Carlton”, registered as early as 1927, was favoured for galanthamine production due to its rich alkaloid content, its wide commercial availability, and its vigour and large size. Like most *Narcissus* cultivars, Carlton is a tetraploid which could be an explanation for its vigour [147]. Unsurprisingly, the two main areas of *Narcissus* cultivation are the United Kingdom and The Netherlands, with breeding of ornamental varieties ongoing since the 16th century. In The Netherlands, the main world producer of ornamental flower bulbs, approximately 20,000 hectares are dedicated to bulb cultivation annually, of which almost 1700 hectares is used for *Narcissus*. These narcissi are primarily grown for ornamental use, with the dwarf cultivar “Tête-à-tête” by far the most popular variety taking up 738 hectares (data 2012/1013 season [148]).

In Asia, the Amaryllidaceae species used for commercial galanthamine production mostly belong to the genus *Lycoris*, also finding its origins in ethnopharmacology and ornamental use. For instance, the species *Lycoris radiata*, or red spider lily, was used in traditional Chinese medicine [149], and is presently used for the commercial production of galanthamine and other alkaloids [150]. Species from this genus are the subject of physiological and molecular research in relation to galanthamine content and its biosynthesis [89,120,151]. In South-America, the rich biodiversity in Amaryllidaceae of this continent is also studied for galanthamine content and domestication potential, and species such as *Habranthus jamesonii* and *Zephyranthes filifolia* are mentioned in this context [152]. These examples show that the choice of which Amaryllidaceae species is being developed for commercial alkaloid production also depends on the region where it is to be cultivated, past traditional medicinal use, and existing expertise of the established ornamental flower bulb industry.

### 5.3. Perspectives on Amaryllidaceae Alkaloid Production

Amaryllidaceae alkaloid production based on *in vitro* grown plant material is being investigated and improved upon, but is to our knowledge not exploited commercially and still considered not economically viable [153,154]. The production of pharmaceutical compounds in heterologous expression systems is much advocated in general, but this may not be the most pragmatic approach for the commercial production of Amaryllidaceae alkaloids. This option is not available for the near future; as such production systems depend on having at least the most essential biosynthetic genes available. Present commercial production of galanthamine is based on the field cultivation of bulbs from species such as *Narcissus* [91]. Given the available traditional expertise in the cultivation and breeding of Amaryllidaceae species for ornamental use, and the existing infrastructure of the flower bulb industry, optimisation of medicinal alkaloid production starting from the existing species and cultivars may well be the most effective route to commercial scale production of new pharmaceutical alkaloids. Similar to the case for opium poppy, as described above, special cultivars each dedicated to the production of specific pharmaceutical alkaloids could be developed, and possibly combined with semi-synthesis of derivatives. Establishing the molecular identity of the biosynthetic genes and understanding their regulation, could significantly speed up the development of such improved cultivars. Amaryllidaceae alkaloid production in heterologous systems could be envisaged for the more distance future, but will have to be economically competitive with established agricultural production systems.

## 6. Conclusions

Galanthamine is presently produced commercially from cultivated Amaryllidaceae as a drug for the symptomatic treatment of Alzheimer’s disease, and other Amaryllidaceae alkaloids are of interest for pharmaceutical drug development. The established ornamental bulb industry has extensive expertise in the cultivation and breeding of Amaryllidaceae species, and can provide a sustainable supply of plant material. Ornamental cultivars from genera such as *Narcissus*, *Leucojum* and *Lycoris* are presently used for the commercial production of galanthamine. Extensive phytochemical and pharmacological analyses have been performed on the Amaryllidaceae and their alkaloids, and biochemical characterisations have resulted in a proposed biosynthetic pathway. In contrast, the molecular identity of the biosynthetic genes is unknown but transcriptome analysis in combination with metabolic profiling are now established procedures enabling gene identification. New experimental approaches of gene identification may also be based on the existence of biosynthetic gene clusters. A molecular understanding of Amaryllidaceae alkaloid biosynthesis will support breeding efforts to produce new cultivars dedicated to alkaloid production, but also pave the way for metabolic engineering and for alkaloid production in heterologous systems. Demographic developments may be an important driver of future commercial Amaryllidaceae alkaloids production.
